# P-2208. Previously Diagnosed, Untreated Hepatitis C Among Emergency Department And Urgent Care Patients

**DOI:** 10.1093/ofid/ofae631.2362

**Published:** 2025-01-29

**Authors:** Vall Vinaithirthan, Kevin Kamis, Emily Hopkins, Shawni Vaughn, Jason Haukoos, Sarah E Rowan

**Affiliations:** University of Vermont, SOUTH BURLINGTON, Vermont; Denver Health and Hospital Authority, Denver, Colorado; Denver Health and Hospital Authority, Denver, Colorado; Denver Health and Hospital Authority, Denver, Colorado; Denver Health and Hospital Authority, Denver, Colorado; Denver Public Health, Denver, CO

## Abstract

**Background:**

Hepatitis C (HCV) incidence has been increasing steadily over the past decade despite the growing availability of well-tolerated, highly effective treatment. Additionally, many individuals who have been living with HCV for decades remain untreated. Emergency departments (EDs) serve large numbers of patients at risk for HCV who commonly do not have access to other forms of care. To address these gaps, innovative approaches to identification and linkage to care are needed. The goal of this study was to assess the number and characteristics of individuals with previously diagnosed but still untreated HCV presenting for acute, unscheduled episodic care, defined as ED or urgent care (UC) visits, in an effort to better understand the potential for HCV care interventions in these settings.

**Methods:**

We performed a retrospective cohort study using electronic health record (EHR) data from Denver Health Medical Center in Denver, Colorado. ED or UC visits between January 1, 2019 and December 31, 2021 among those aged ≥ 18 years with evidence of HCV viremia at any time between January 1, 2016 and the day prior to the visit were included. We used descriptive statistics to characterize patient- and visit-level characteristics, timing of visits, and evidence of HCV clearance afterward.

**Results:**

Over the 3-year study period, 1,407 unique individuals with evidence of current HCV infection were seen in the ED or UC, representing 5,829 total visits. At the patient level, the median age was 51 years (IQR 38-60), 53% were white, non-Hispanic, 29% Hispanic, and 73% cis-gender male. At the visit level, 17% were triaged as emergent status, 60% as urgent, and 16% less urgent. Visits were distributed relatively evenly throughout the week with Thursday being the most common day for qualifying visits (16%). Median length of stay for visits that did not lead to hospitalization was 276 minutes (IQR 153-461). Medicaid was the payer source for 73% and the most common chief complaints were related to substance use (18%) and pain (18%).

**Conclusion:**

Substantial numbers of patients with previously diagnosed but untreated HCV are seen in the ED and UC annually, many of whom have Medicaid coverage. These healthcare locations may be ideal venues for addressing HCV through identification, referral to care services, and potentially treatment.

**Disclosures:**

All Authors: No reported disclosures

